# Birth and newborn care policies and practices limit breastfeeding at maternity facilities in Vietnam

**DOI:** 10.3389/fnut.2022.1041065

**Published:** 2022-10-28

**Authors:** Tuan T. Nguyen, Jennifer Cashin, Hoang T. Tran, Tuan A. Hoang, Roger Mathisen, Amy Weissman, John C. S. Murray

**Affiliations:** ^1^Alive & Thrive East Asia Pacific, FHI 360, Hanoi, Vietnam; ^2^Alive & Thrive East Asia Pacific, FHI 360, Washington, DC, United States; ^3^Neonatal Unit and Human Milk Bank, Department of Pediatrics, School of Medicine and Pharmacy, Da Nang Hospital for Women and Children, The University of Da Nang, Da Nang, Vietnam; ^4^Department of Maternal and Child Health, Vietnam Ministry of Health, Hanoi, Vietnam; ^5^Asia Pacific Regional Office, FHI 360, Bangkok, Thailand; ^6^Independent Researcher, Iowa City, IA, United States

**Keywords:** early essential newborn care, early initiation breastfeeding, exclusive breastfeeding, maternity facilities, newborn, policy, the Code, Vietnam

## Abstract

The prevalence of early and exclusive breastfeeding in Vietnam remains sub-optimal. The objective of this study was to determine factors associated with early initiation of breastfeeding (EIBF) and exclusive breastfeeding for the first 3 days after birth (EBF3D). We conducted a population-based, cross-sectional survey of 726 mothers with children aged 0–11 months in two provinces and one municipality from May to July 2020. Multinomial logistic regression was used to examine factors associated with EIBF and EBF3D. The prevalence of EIBF was 39.7% and EBF3D 18.0%. The EIBF prevalence is positively associated with immediate and uninterrupted skin-to-skin contact (SSC) for 10–29 min (aOR: 2.55; 95% CI: 1.49, 4.37), 30–59 min (aOR: 4.15; 95% CI: 2.08, 8.27), 60–80 min (aOR: 4.35; 95% CI: 1.50, 12.6), or ≥90 min (aOR: 5.87; 95% CI: 3.14, 10.98). EIBF was negatively associated with cesarean birth (aOR: 0.24; 95% CI: 0.11, 0.51), bringing infant formula to the birth facility (aOR: 0.49; 95% CI: 0.30, 0.78), purchased it after arrival (aOR: 0.37; 95% CI: 0.24, 0.60), or did both (aOR: 0.43; 95% CI: 0.21, 0.89). EBF3D was negatively associated with cesarean section birth (aOR: 0.15; 95% CI: 0.06, 0.39), vaginal birth with episiotomy (aOR: 0.40; 95% CI: 0.18, 0.88), bringing formula to the maternity facility (aOR: 0.03; 95% CI: 0.01, 0.07), purchased it after arrival (aOR: 0.02; 95% CI: 0.01, 0.06) or did both (aOR: 0.04; 95% CI: 0.02, 0.10). Receiving counseling from any source was not significantly associated with early breastfeeding practices. Policy and health service delivery interventions should be directed at eliminating infant formula from birthing environments, reducing unnecessary cesarean sections and episiotomies, providing immediate and uninterrupted SSC for all births, and improving breastfeeding counseling and support.

## Introduction

Breastfeeding is nature’s perfect food system and the biological norm for feeding human infants and young children (1). Early initiation of breastfeeding (EIBF) within the first hour of life and exclusive breastfeeding (EBF) up to 6 months are associated with reduced child morbidity and mortality and provide long-term benefits for both mother and child (2–4). Although almost all mothers are biologically capable of breastfeeding their children (5) and 95% of babies globally receive some breastmilk, breastfeeding practices remain sub-optimal (6). Countries in East Asia and the Pacific have lower prevalence rates of EIBF (38%), exclusive breastfeeding for the first 2 days after birth (57%), and EBF up to 6 months (31%) compared to corresponding global rates of 48, 65, and 44% (7, 8). In Vietnam, rates of EIBF fell nationally between 2011 and 2020 from 39.7 to 23.5% (9, 10); and bottle-feeding rates rose (from 38.7 to 54.3%) (9, 10). Further, the cesarean section rate rose from 20.0% nationally in 2011 to 33.4% in 2020, with rates in some cities above 50.0% (9–11), a trend that is likely to further reduce the likelihood of breastfeeding (12–15).

These declines have occurred in Vietnam despite efforts since the 1990s to put in place policies to protect, promote, and support breastfeeding (16, 17). A number of policies and regulations have been adopted, including: national legislation on the Code of Marketing of Breast milk Substitutes (“the Code”) (18–20); public and private hospital accreditation standards that promote the Ten Steps to Successful Breastfeeding (21); early essential newborn care practice standards for vaginal and cesarean births, a package of evidence-based interventions applied in the second stage of labor and early newborn period to improve maternal and newborn outcomes, including early and exclusive breastfeeding (22–25); policies promoting breastfeeding counseling and support from pregnancy through the first 2 years of life (25); and designation criteria and assessment mechanisms for Centers of Excellence for Breastfeeding (26).

Early initiation of breastfeeding and EBF for 6 months are associated with several individual factors including maternal education, type of work, parity, smoking, race, and ethnicity; as well as practices during and immediately following childbirth (27–29). Health service-related factors around childbirth known to influence breastfeeding include cesarean birth, episiotomy, immediate and uninterrupted skin-to-skin contact (SSC) of adequate duration, rooming-in of mother and newborn, and breastfeeding counseling (27–29). EIBF and EBF at hospital discharge are associated with EBF up to 6 months and continued breastfeeding (30–34). Aggressive marketing of commercial milk formula for infants, children, and pregnant women impedes breastfeeding and the provision of breastfeeding support by health workers (27–29, 35–38).

Given Vietnam’s high institutional birth rate (96.3% in 2020) (10), health facility environments and health worker behaviors play important roles in influencing early and exclusive breastfeeding around the time of birth. Adoption of early essential newborn care protocols in studies of maternity hospitals in Vietnam has been associated with improved early and exclusive breastfeeding practices prior to discharge (24, 34). However, there are limited data on how widely these protocols are being implemented. Additionally, the impact of factors around childbirth on post-discharge breastfeeding practices has not been documented in a population-based sample of women. Population-based data from a study investigating breastfeeding promotion, protection and support in Vietnam provided an opportunity to examine factors associated with early and exclusive breastfeeding in a representative sample of Vietnamese women (39). The primary objective of this study was to identify maternal and health system factors, including antenatal care and birth practices, which were associated with early and exclusive breastfeeding in the first 3 days after birth. The goal was to use these findings to identify policy and program interventions to address priority barriers to improve breastfeeding practices in Vietnam.

## Materials and methods

### Sample and data sources

Primary data collection was conducted for a population-based, cross-sectional study reviewing the content, implementation, and potential impact of policies to protect, promote, and support breastfeeding in Vietnam. The design was a population-based, cross-sectional observational survey using both quantitative and qualitative methods. Data were collected between May and July 2020. Details of study design, sampling and data collection tools (Supplementary material) were presented in a research protocol (39) published prior to the data analysis.

The sampling frame included two provinces and one municipality selected to be representative of the different socio-economic characteristics of Vietnam: Bac Ninh, a province that is transforming from a predominantly agricultural to a more industrialized province in Red River Delta Region (north), with an estimated population of 1,380,000 of which 28% is urban; Binh Duong, a predominantly industrial province in the Southeastern Region (south) with an estimated population of 2,460,000 of which 80% is urban; and Ho Chi Minh City (HCMC), the most populous city in Vietnam (south), with an estimated population of 9,040,000 of which 80% is urban (40). In 2019, there were about 34,200 live births in Bac Ninh, 43,200 in Binh Duong, and 127,400 in HCMC (40).

A stratified multiple-stage cluster sampling design was used to obtain an estimated minimum sample size of 620 mothers of children 0–11 months (38, 39). Within each study location, all sub-districts were divided into three categories: industrial zone, urban without an industrial zone, and rural without an industrial zone; in each category, one district was randomly sampled. Within each sampled district all sub-districts were listed, and ten sub-districts randomly selected; and within each sub-district all mothers of infants aged 0–11 months were listed using immunization records provided by community health workers. Mothers of infants aged 0–11 months were then selected using systematic random sampling. Because the lists used for sampling were from immunization records, the sample included both permanent and temporary residents (i.e., migrant workers) of the selected sub-districts.

Health workers contacted selected mothers and invited them to participate. If the sampled woman was unable or unwilling to participate in the survey, she was replaced by another woman randomly selected from the sub-district list. The non-response rate was 14.6%. Those who agreed to participate were contacted by the research coordinators who arranged a time for a household visit (38, 39). Evaluators visited sampled households in pairs and obtained written consent from all participants. All interviews were conducted using a structured questionnaire in Vietnamese by a team of two trained supervisors in a private and quiet place with the mother of the child alone (i.e., without the presence of father, grandmother, or other caregivers). Quantitative data from mothers were collected electronically using tablets and uploaded daily to a secure cloud-based server. A data manager downloaded the data from the cloud-based server and conducted frequent data quality checks (39).

### Definition of variables

#### Dependent variables

The main outcome variables for this analysis were EIBF and exclusive breastfeeding during the first 3 days after birth (EBF3D) (41, 42) among infants <12 months, which can be affected by early practices and facility support around birth. EIBF was defined as infants who were put to the breast for the first time within 1 h of birth (41, 42). The mother was asked “How soon after birth did you put (NAME) to the breast for the first time?” and if she responded with a time less than 1 h, was defined as practicing EIBF.

Exclusive breastfeeding during the first 3 days after birth was defined as infants who were fed exclusively with breast milk for the first 3 days after birth. The interviewers asked the mothers whether their infant had received any of the following in the first 3 days of birth: breastmilk, breastmilk from another woman, infant formula/other infant milk, plain water, honey, sugar or glucose water, lemon juice/herbal tea (e.g., licorice root), and any other food or drink. A mother who responded yes to human milk and no to any other food or drink was defined as practicing EBF3D.

#### Independent variables

Independent variables for analysis were selected if they were collected by the survey data collection tools and had been associated or potentially associated with breastfeeding outcomes in the World Health Organization (WHO) data synthesis reviews developed using the Grading of Recommendations Assessment, Development and Evaluation methodology (32, 43), namely: antenatal care (ANC) contacts, mode of birth (cesarean section, vaginal with or without episiotomy), SSC, breastfeeding before separation, rooming-in of mother and newborn, intention to use infant formula in the perinatal period, distribution of infant formula samples during the facility stay, and several sociodemographic characteristics.

*Birth mode* was defined by using two questions: “Did you have a cesarean section when you gave birth to (NAME)?” and for those who had vaginal birth “Did you have an episiotomy when you gave birth to (NAME)?” Respondents were then grouped into one of three mutually exclusive categories based on their responses: (1) Vaginal birth without episiotomy, (2) Vaginal birth with episiotomy, and (3) Cesarean birth.

*Skin-to-skin contact* was defined by using three questions: “After giving birth, was (NAME) placed on your chest skin to skin?,” “How long did it take from (NAME)’s first cry until (he/she) was put onto your chest?,” and “For how long was (NAME) kept skin to skin on your chest with you with no break or separation?” Respondents were then grouped into one of six mutually exclusive categories based on their responses: (1) Not applied or applied after 1 min and (2) applied within 1 min and uninterrupted for (a) <10 min, (b) 10–29 min, (c) 30–59 min, (d) 60–89 min, and (e) at least 90 min (29, 32).

*Intention to use infant formula during the perinatal period* was indirectly defined by using two questions: “Did you or your family member bring any infant formula to the health facility when you gave birth to (NAME)?” and “Did you or your family member purchase any infant formula at or near the health facility shortly after you gave birth to (NAME)?” Respondents were then grouped into one of four mutually exclusive categories based on their responses: (1) Did not bring or purchase, (2) Brought, (3) Purchased, and (4) Brought and purchased (38).

*Antenatal care* received was defined by both type of provider and number of care visits received during pregnancy. Women were asked whether they visited public or private facilities for each ANC visit and the type of facility categorized into public health facilities only, both public and private health facilities, and private health facilities only. The number ANC visits made between the beginning of pregnancy and birth was categorized into 0–3, 4–7, and at least 8 times (25, 44).

*Birthweight* was reported by the mother, and newborns weighing less than 2,500 g were classified as low birth weight.

*Gestational age* was defined as the number of weeks of pregnancy reported by the mother at the time of the birth of their baby; babies born at less than 37 weeks of gestation were defined as preterm (45). The number of previous child(ren) was identified by subtracting one from the total number of child(ren) the mother had. It was then categorized into zero (first child), one and two or more. In addition, we included the sex of the child (male or female) in the analysis.

*Advice received on breastfeeding* during pregnancy and in the first 3 days after birth by a health worker or lay person was defined as any verbal information, counseling, observation and breastfeeding assessment with feedback or any other information received, as reported by the mother.

*Socio-economic characteristics* of participating women included age (years), ethnicity (Kinh, the ethnic majority group in Vietnam, and other ethnicities), marital status (married or unmarried), education (never attended school, primary school, junior secondary school, secondary school, diploma or postgraduate), and employment status (farmer, blue-collar, white-collar, small trader or self-employed, and unemployed, homemaker, student or other) (38).

### Data analysis

Data analysis was performed using Stata 15.1 (Stata Inc., College Station, TX, USA). For descriptive analysis, we analyzed general characteristics, experience during ANC and feeding practices in the first 3 days after birth. We conducted multinomial logistic regression to examine associations between exposure variables and EIBF and EBF3D controlled for potential confounding factors and adjusted for clustering [e.g., province or municipality and the 30 primary sampling units (PSUs) within each province] by using the robust option. We neither estimated sampling weights nor used them in the analysis because our primary focus was on the assessment of association rather than the estimation of prevalence (39). For EBF3D, in addition to variables included in the regression model for EIBF, we included the following variables: EIBF, completion of the first breastfeed before separation, rooming-in, and receipt of a free infant formula sample during hospital stay.

## Results

A total of 726 interviews with mothers with infants aged 0–11 months (infants) were completed. Of the 726 mothers, 95.3% were of Kinh ethnicity; and the remaining ethnicities were Hoa (1.1%), Khmer (1.1%), Muong (0.8%), Nung (0.7%), Tay (0.6%), Cham (0.1%), Tho (0.1%), and Xtieng (0.1%) (Table 1). Ninety-nine percent (98.9%) of mothers were married, 62.8% had a secondary diploma or higher, and 23.1% had a white-collar job (Table 1).

**TABLE 1:** Socio-economic characteristics of mothers of infants 0–11 months, three provinces, Vietnam 2020^1^.

	HCMC (*n* = 243)	Binh Duong (*n* = 241)	Bac Ninh (*n* = 242)	Total (*n* = 726)
Kinh ethnicity	95.1	95.4	95.5	95.3
Age (Mean ± SD; median, p25–p75)	30.7 ± 5.7 31 (27–35)	29.5 ± 5.3 30 (26–33)	29 ± 5.3 29 (25–33)	29.7 ± 5.5 29 (26–34)
Marital status, Married	97.9	99.2	99.6	98.9
**Highest level of education:**				
Primary school or less	18.9	18.3	8.7	15.3
Junior secondary school	26.3	24.5	14.9	21.9
Secondary school	25.5	24.1	30.2	26.6
Diploma, bachelor, or higher	29.2	33.2	46.3	36.2
**Main occupation:**				
Blue-collar or farmer	28.4	35.7	30.6	31.5
White-collar	18.5	22.0	28.9	23.1
Small trader, self-employed, small self-owned business, services	30.5	18.7	32.6	27.3
Unemployed, homemaker, student	22.6	23.7	7.9	18.3

^1^Data presented as % except for age presented as mean ± Standard Deviation (SD) and median and interquartile range. HCMC, Ho Chi Minh City.

While most mothers in our sample (98.9%) report breastfeeding their newborns during the first 3 days of life, only 39.7% initiated breastfeeding in the first hour after birth (Table 2). Mothers from HCMC were more likely to report EIBF (53.1%) while those from Bac Ninh were least likely (30.2%) (Table 2). During the first 3 days of life, 90.8% of mothers fed their newborns with their own breastmilk, 1.1% with breastmilk from other mothers (either donor human milk from a milk bank or wet nursing), and 3.3% from both (Table 2).

**TABLE 2:** Feeding practices in the first 3 days after birth reported by mothers of infants 0–11 months, three provinces, Vietnam, 2020^1^.

	HCMC (*n* = 243)	Binh Duong (*n* = 241)	Bac Ninh (*n* = 242)	Total (*n* = 726)
Early initiation of breastfeeding	53.1	35.7	30.2	39.7
Exclusive breastfeeding for the first 3 days after birth	23.5	23.7	7.0	18.0
**Food and drink given in the first 3 days after birth:**				
**Human milk:**				
Any	93.4	92.5	89.7	91.9
Mothers’ own milk	93.0	90.5	88.8	90.8
Milk from another mother	3.3	3.3	6.6	4.4
Infant formula/other infant milk	72.0	73.4	92.6	79.3
Plain water	20.6	21.2	7.4	16.4
Honey	4.9	4.6	0.4	3.3
Sugar or glucose water	0.8	1.2	0.0	0.7
Lemon juice/herbal tea (e.g., licorice root)	0.0	0.4	0.0	0.1

^1^Data presented as %. HCMC, Ho Chi Minh City.

Less than one fifth (18.0%) of mothers reported EBF3D: the prevalence was higher in Binh Duong and HCMC at 23.7 and 23.5%, respectively, than in Bac Ninh (7.0%) (Table 2). Infant formula was the most common supplement provided to newborns in the first 3 days of life, with 79.3% of mothers across the three provinces (72.0% in HCMC, 73.4% in Binh Duong, and 92.6% in Bac Ninh) reporting that they provided infant formula to their newborns during this period. Provision of plain water (16.4%) was the second most common supplement, which was given on its own in 2.1% of cases and used to mix formula for the remainder (Table 2). Provision of honey (3.3), sugar water (0.7%), and lemon juice (0.1%) was far less common (Table 2).

Sampled women were more likely to seek antenatal care ANC in a private health facility (35.5%) and a mix of public and private facilities (35.5%) than public facilities (24.2%); and 71.3% of mothers reported four or more ANC visits (Table 3). Nearly three quarters of respondents (72.6%) reported consuming commercial milk formula for pregnant women at least once during their pregnancy (Table 3), with a similar prevalence across provinces. About two thirds of respondents received breastfeeding guidance from a health worker in a health facility, with the highest prevalence in HCMC followed by Binh Duong, and lowest in Bac Ninh (Table 3). Nearly two thirds (59.1%) received breastfeeding advice from another person, mainly mothers or mothers-in-law, husband, other family members, neighbors, friends, and co-workers (Table 3). In our sample, 28.5% of mothers were first-time mothers (Table 3).

**TABLE 3:** Experience during pregnancy reported by the mothers of infants 0–11 months, three provinces, Vietnam, 2020^1^.

	HCMC (*n* = 243)	Binh Duong (*n* = 241)	Bac Ninh (*n* = 242)	Total (*n* = 726)
**Places of antenatal care visits:**				
Public hospital only	37.4	28.2	7.0	24.2
Both public and private hospital or clinic	25.5	29.5	51.7	35.5
Private hospital or clinic only	37.0	42.3	41.3	40.2
**The number of antenatal care visits**				
0–3 times	6.2	7.5	56.6	23.4
4–7 times	21.4	17.8	25.2	21.5
≥8 times	72.4	74.7	18.2	55.1
Used commercial milk formula for pregnant women	68.7	79.7	69.4	72.6
Received breastfeeding advice by a health worker from a health facility	81.1	67.2	50.4	66.3
**Received breastfeeding advice by another person:**	65.8	62.2	49.2	59.1
Mother or mother-in-law	56.0	53.5	39.3	49.6
Husband	22.6	21.6	9.9	18.0
Other family members	24.3	37.3	19.4	27.0
Neighbors, friends, or co-workers	23.0	24.9	21.1	23.0
Nutrition collaborator	0.8	0.0	0.0	0.3
Hamlet health worker	0.4	0.0	2.9	1.1
Women union staff	0.4	0.0	0.0	0.1
**Number of the previous child(ren)**				
Zero (this was the first child)	30.5	32.4	22.7	28.5
1	52.3	47.7	39.7	46.6
≥2	17.3	19.9	37.6	24.9

^1^Data presented as %. HCMC, Ho Chi Minh City.

Eighty-nine percent of women gave birth in public health facilities (59.2% in a public hospital; 29.5% in a public polyclinic) (Table 4). A high proportion of newborns were born at term (94.2%), heavier than 2,500 g (95.3%), and birthed by cesarean (44.6%) or vaginally with an episiotomy (46.1%); 9.2% were birthed vaginally without an episiotomy. Birth practices were similar across provinces. Overall, 49.3% of mothers reported immediate (within 1 min after birth) SSC, with 27.8 receiving uninterrupted SSC for less than 30 min and 11.6% receiving uninterrupted SSC for the recommended 90 min (Table 4). The prevalence of immediate and prolonged SSC for 90 min was highest in HCMC (18.1%), then Binh Duong (16.2%) and lowest in Bac Ninh (0.4%) (Table 4).

**TABLE 4:** Experience during the perinatal period reported by mothers of infants 0–11 months, three provinces, Vietnam 2020^1^.

	HCMC (*n* = 243)	Binh Duong (*n* = 241)	Bac Ninh (*n* = 242)	Total (*n* = 726)
**Birthplace:**				
Public hospital (provincial and central levels)	61.7	65.1	50.8	59.2
Public polyclinic, district health center	26.3	18.7	43.4	29.5
Private hospital	11.9	16.2	5.8	11.3
**Sex of newborn**				
Female	51.0	47.7	43.0	47.2
Male	49.0	52.3	57.0	52.8
**Birthweight:**				
Birthweight of 2,500 g or heavier	95.9	93.8	96.3	95.3
Birthweight (g) Mean ± SD, median (p25–p75)	3,185 ± 428 3,200 (2,900–3,450)	3,163 ± 446 3,200 (2,900–3,450)	3,250 ± 418 3,200 (3,000–3,500)	3,199 ± 432 3,200 (2,900–3,500)
**Gestational age:**				
Gestational age of 37 weeks or longer	92.2	95.4	95.0	94.2
Gestational age (weeks) Mean ± SD, median (p25–p75)	38.6 ± 1.4 39 (38–40)	38.8 ± 1.6 39 (38–40)	38.9 ± 1.3 39 (38–40)	38.7 ± 1.4 39 (38–40)
**Birth mode:**				
Vaginal birth without episiotomy	11.5	5.8	10.3	9.2
Vaginal birth with episiotomy	42.8	50.6	45.0	46.1
Cesarean birth	45.7	43.6	44.6	44.6
**Skin-to-skin contact:**				
None or later than 1 min	48.1	47.7	56.2	50.7
Within 1 min any duration	51.9	52.3	43.8	49.3
<10 min	8.6	8.7	21.9	13.1
10–29 min	14.0	14.1	16.1	14.7
30–59 min	7.0	9.1	5.0	7.0
60–89 min	4.1	4.1	0.4	2.9
≥90 min	18.1	16.2	0.4	11.6
Completed first breastfeed before being separated from mothers	46.5	30.3	12.8	29.9
Rooming-in	54.7	57.7	85.5	66.0
**Brought or purchased infant formula by the health facility of birth:**				
Did not bring or purchase	17.3	14.9	4.1	12.1
Brought	45.3	24.9	64.5	44.9
Purchased	31.3	55.6	19.4	35.4
Brought and purchased	6.2	4.6	12.0	7.6
Received free infant formula sample during the hospital stay	7.0	7.5	3.3	5.9
Received breastfeeding advice by a health worker during the hospital stay	70.0	59.3	36.4	55.2
**Received breastfeeding advice by another person during the hospital stay:**	30.5	19.9	30.6	27.0
Mother or mother-in-law	25.1	17.4	26.9	23.1
Husband	0.8	0.4	0.8	0.7
Other family members	9.1	2.5	6.6	6.1
Neighbors, friends, or co-workers	2.1	1.2	1.2	1.5
Nutrition collaborator	0.4	0.0	0.0	0.1
Hamlet health worker	0.4	0.0	0.0	0.1
Women union staff	0	0	0	0

^1^Data presented as % except for birthweight and gestational age presented as mean ± SD.

**FIGURE 1:**
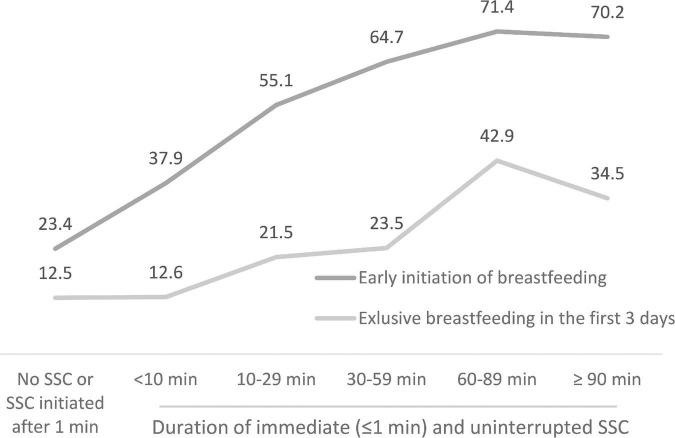
Skin-to-skin contact (SSC) and breastfeeding practices reported by mothers of infants 0–11 months, three provinces, Vietnam, 2020.

The prevalence of EIBF showed an increased trend with the duration of SSC, rising from 23.4% among newborns who did not receive immediate SSC to around 70% among those who received immediate and uninterrupted SSC for at least 90 min (Figure 1). The prevalence of EBF3D also showed an increased trend with the duration of SSC, rising from 12.5% among newborns who did not receive immediate SSC to 42.9% among those receiving 60–89 min of uninterrupted SSC, and 34.5% of those receiving over 90 min (Figure 1).

A third (29.9%) of newborns completed their first breastfeed before being separated from their mothers and 66.0% stayed with their mothers from birth (rooming-in) (Table 4). Many mothers (87.9%) reported bringing infant formula or buying it near the maternity facility; and 5.9% reported receiving an infant formula sample during their stay at the facility (Table 4). Just over half (55.2%) of mothers reported receiving breastfeeding counseling and support from a health worker during their hospital stay. More mothers in HCMC received breastfeeding support by a health worker during the hospital stay (70.0%) than Binh Duong (59.3%), and Bac Ninh (36.4%) (Table 4). About one in four mothers (27.0%) received breastfeeding advice from another person, mainly from mothers or mothers-in-law during the first 3 days after birth (Table 4).

Multinomial logistic regression showed that women who received ANC from a mix of both public and private facilities were significantly less likely (aOR: 0.59; 95% CI: 0.40, 0.87) to initiate breastfeeding within the first hour than those who received ANC at public health facilities only (Table 5). The likelihood of EIBF was significantly lower among those who gave birth via cesarean (aOR: 0.24; 95% CI: 0.11, 0.51), brought infant formula to the maternity facility (aOR: 0.49; 95% CI: 0.30, 0.78), purchased it after arrival (aOR: 0.37; 95% CI: 0.24, 0.60), or did both (aOR: 0.43; 95% CI: 0.21, 0.89) (Table 5). Mothers were less likely to practice EIBF in Binh Duong (aOR: 0.42; 95% CI: 0.22, 0.83) and Bac Ninh (aOR: 0.44; 95% CI: 0.21, 0.94) than those from HCMC. EIBF was significantly more likely if immediate and uninterrupted SSC was applied for 10–29 min (aOR: 2.55; 95% CI: 1.49, 4.37), 30–59 min (aOR: 4.15; 95% CI: 2.08, 8.27), 60–80 min (aOR: 4.35; 95% CI: 1.50, 12.6), or ≥90 min (aOR: 5.87; 95% CI: 3.14, 10.98). In addition, women who had one child (aOR: 1.96; 95% CI: 1.26, 3.04) or two or more children (aOR: 2.41; 95% CI: 1.26, 4.61) were significantly more likely to practice EIBF than mothers giving birth to their first child (Table 5). No other variables showed significant relationships with EIBF.

**TABLE 5:** Multinomial logistic regression analysis of the relationship between key variables and breastfeeding practices reported by mothers of infants 0–11 months, three provinces, Vietnam 2020^a^.

	Early initiation of		Exclusive breastfeeding for the	
	breastfeeding (*n* = 726)		first 3 days after birth (*n* = 726)	
	aOR	95% CI	aOR	95% CI
**Place of antenatal care visits:**				
Public hospital only	1.00	(1.00, 1.00)	1.00	(1.00, 1.00)
Both public and private hospital/clinic	0.59**	(0.40, 0.87)	0.89	(0.45, 1.74)
Private hospital only	0.68	(0.44, 1.05)	1.23	(0.69, 2.17)
**The number of antenatal care visits**				
0–3 times	1.00	(1.00, 1.00)	1.00	(1.00, 1.00)
4–7 times	1.03	(0.61, 1.74)	0.74	(0.38, 1.42)
≥8 times	1.79	(0.86, 3.72)	1.27	(0.46, 3.53)
Received breastfeeding advice during antenatal visits from a facility-based health worker	1.36	(0.95, 1.95)	1.30	(0.63, 2.69)
**Newborn characteristics**				
**Sex of newborn**				
Female	1.00	(1.00, 1.00)	1.00	(1.00, 1.00)
Male	1.30	(0.92, 1.83)	1.00	(0.58, 1.73)
Birthweight of 2,500 g or heavier	2.40	(0.73, 7.87)	1.04	(0.26, 4.17)
Gestation age of 37 weeks or longer	1.15	(0.53, 2.48)	1.23	(0.62, 2.46)
**Birth mode:**				
Vaginal birth without episiotomy	1.00	(1.00, 1.00)	1.00	(1.00, 1.00)
Vaginal birth with episiotomy	0.75	(0.39, 1.46)	0.40*	(0.18, 0.88)
Cesarean birth	0.24***	(0.11, 0.51)	0.15***	(0.06, 0.39)
**Skin-to-skin contact:**				
None or later than 1 min	1.00	(1.00, 1.00)	1.00	(1.00, 1.00)
<10 min	1.42	(0.75, 2.67)	0.61	(0.20, 1.85)
10–29 min	2.55***	(1.49, 4.37)	0.69	(0.29, 1.63)
30–59 min	4.15***	(2.08, 8.27)	1.19	(0.48, 3.00)
60–89 min	4.35**	(1.50, 12.60)	1.04	(0.28, 3.88)
≥90 min	5.87***	(3.14, 10.98)	1.35	(0.43, 4.23)
**Brought or purchased infant formula at the health facility at the time of birth:**				
Did not bring or purchase	1.00	(1.00, 1.00)	1.00	(1.00, 1.00)
Brought	0.49**	(0.30, 0.78)	0.03***	(0.01, 0.07)
Purchased	0.37***	(0.24, 0.60)	0.02***	(0.01, 0.06)
Brought and purchased	0.43*	(0.21, 0.89)	0.04***	(0.02, 0.10)
Received breastfeeding advice after birth from a facility-based health worker	1.47	(0.92, 2.34)	1.39	(0.75, 2.59)
**Number of the previous child(ren)**				
Zero (this was the first child)	1.00	(1.00, 1.00)	1.00	(1.00, 1.00)
1	1.96**	(1.26, 3.04)	1.48	(0.71, 3.08)
≥2	2.41**	(1.26, 4.61)	1.80	(0.60, 5.33)
**Highest level of education:**				
Primary school or less	0.69	(0.36, 1.32)	0.22**	(0.09, 0.56)
Junior secondary school	0.87	(0.45, 1.69)	1.51	(0.57, 3.95)
Secondary school	1.31	(0.78, 2.20)	1.08	(0.52, 2.27)
Diploma, bachelors or higher	1.00	(1.00, 1.00)	1.00	(1.00, 1.00)
**Place of residence:**				
Ho Chi Minh City	1.00	(1.00, 1.00)	1.00	(1.00, 1.00)
Binh Duong	0.42*	(0.22, 0.83)	1.43	(0.81, 2.51)
Bac Ninh	0.44*	(0.21, 0.94)	0.48	(0.20, 1.15)
Early initiation of breastfeeding			1.47	(0.76, 2.85)
Completed first breastfeed before being separated from mothers			1.26	(0.66, 2.43)
Rooming-in			1.12	(0.62, 2.00)
Received free commercial milk formula sample during the hospital stay			0.12**	(0.02, 0.55)

^a^Data from the Code impact study in Vietnam in 2020. Values are adjusted odds ratios (aOR) and 95% Confidence Intervals (95% CI) from Multinomial logistic regression, controlled for use of commercial milk formula for pregnant women, received breastfeeding advice from lay person during antenatal visits, type of birthplace (public/private health facility), received breastfeeding advice from a lay person after birth, mothers’ ethnicity, age, and main occupation. We used robust option to account for clustering. Significantly different from the null value (aOR = 1; two-sided *t*-tests): *p < 0.05, **p < 0.01, ***p < 0.001.

Exclusive breastfeeding during the first 3 days after birth was less likely among mothers who gave birth by cesarean section (aOR: 0.15; 95% CI: 0.06, 0.39), had a vaginal birth with episiotomy (aOR: 0.40; 95% CI: 0.18, 0.88), brought infant formula to the maternity facility (aOR: 0.03; 95% CI: 0.01, 0.07), purchased it after arrival (aOR: 0.02; 95% CI: 0.01, 0.06) or did both (aOR: 0.04; 95% CI: 0.02, 0.10) (Table 5). Women who had a primary school or less education were less likely to practice EBF3D (aOR: 0.22; 95% CI: 0.09, 0.56), as were those who received a free infant formula sample during their hospital stay (aOR: 0.12; 95% CI: 0.02, 0.55) (Table 5). No other variables showed significant relationships with the prevalence of EBF3D.

## Discussion

This study of 726 mothers with infants aged 0–11 months from provinces representative of the socio-economic characteristics of Vietnam found a low prevalence of EIBF (39.7%) and EBF3D (18.0%), and high prevalence of infant formula use (79.3%). Rates of cesarean birth (44.6%) and vaginal births with episiotomy (46.1%) were high. Forty-nine percent of mothers received immediate SSC after birth and 11.6% uninterrupted SSC for the recommended duration of 90 min or more.

In univariate analysis both EIBF and EBF3D showed an increased trend with the duration of SSC, with prolonged SSC of 90 min associated with an EIBF prevalence of 70% and EBF3D of 34%. Eighty-eight percent of mothers brought formula to the birth hospital or purchased it after arrival. All women in this population received ANC, with 55% receiving at least eight contacts and most gave birth at public health facilities. Most babies were normal birthweight and term. We found that EIBF was significantly less likely among women who received ANC at both public and private facilities, gave birth by cesarean, brought formula to the birth facility, or purchased it after arrival, or lived in Binh Duong province. EIBF was 2.49–5.66 times more likely if immediate and uninterrupted SSC was applied 10–90 min after birth; and among women with one or more children. EBF3D was significantly less likely among mothers who gave birth by cesarean section, had a vaginal birth with episiotomy, brought formula to the birth facility or purchased it after arrival, and who received a free infant formula sample during their hospital stay. Mothers who had a primary school or less education were significantly less likely to practice EBF3D.

The association of cesarean birth and episiotomy with reduced likelihood of early and exclusive breastfeeding is consistent with previous studies (12, 13, 33, 46, 47). Cesarean births are well recognized as a barrier to successful breastfeeding for a number of reasons including: early separation of newborns to neonatal intensive care units (NICUs) or nurseries for observation (24), staff concerns that immediate SSC or breastfeeding will compromise the procedure, reduce the safety of mothers and babies, or add to their work burden; physical organization of the operating room; lack of coordination and communication between anesthesiology and obstetrics staff; and the belief that maternal pain after the procedure makes breastfeeding difficult (48, 49). However, data from Vietnam and elsewhere show that SSC can be introduced successfully with cesarean section, is safe for both mothers and newborns, and can decrease NICU admissions, improve newborn outcomes, increase maternal satisfaction and exclusive breastfeeding rates (31, 50–52). Similarly, pain following episiotomy may limit the comfort of the mother and contribute to reported breastfeeding difficulties (46).

The significant association between EIBF and the duration of immediate and uninterrupted SSC is consistent with a previous study of in eight countries in Asia and the Pacific, including Vietnam (29). This association remains significant even in the presence of remarkably high rates of cesarean birth, episiotomy, and formula availability at birth facilities and after controlling for low birthweight and preterm birth. Readiness to breastfeed is highly variable between newborns, with the mean time of the first breastfeed around 50 min postpartum. Because a high proportion of mothers require well over 1 h to complete feeding, longer periods of uninterrupted SCC allow this process to be completed (50). SSC promotes thermoregulation, early and exclusive breastfeeding, bonding, reduced stress, earlier expulsion of the placenta and reduced risk of bleeding in the mother among other benefits (31, 50, 51). By preventing separation, newborns are further protected from the negative consequences of harmful procedures including early cord clamping, routine suction, and early bathing, which may slow down the readiness to breastfeed and have other negative health impacts (53). SSC is often interrupted for routine care, including weighing and administration of vitamin K and vaccines, procedures that can be delayed until after 90 min, and which may further interfere with early breastfeeding (50). During the COVID-19 pandemic, provision of SSC has been further negatively impacted by inappropriate national and international guidance recommending the separation of mothers and newborns to prevent disease transmission (54). We found that mothers in HCMC were more likely to practice EIBF. This may suggest better implementation of early essential newborn care standards in facilities in this province and therefore better facility staff preparation, supportive environments, and counseling practices. Since Tu Du Hospital, an early implementer of early essential newborn care is present in HCMC and led hospital coaching in the South of Vietnam, including in Binh Duong province, this association may explain improved breastfeeding practice at birth in southern hospitals (34).

We did not find a statistically significant association between EBF3D and either EIBF or early and uninterrupted SSC. It has been noted previously in population studies in Vietnam that EIBF may not lead to EBF3D (33, 47). However, this finding is not consistent with data from several studies that have demonstrated SSC to be associated with increased likelihood of exclusive breastfeeding from hospital discharge up to 6 months post birth (31). Data from early essential newborn care implementing hospitals in Vietnam have shown that SSC is associated with increased likelihood of exclusive breastfeeding at discharge in hospitals that have conducted staff clinical coaching and upgraded environments to support SSC and early breastfeeding using quality processes (24, 29, 52). It seems therefore that provider behaviors (e.g., unnecessary medical procedures and separation of mothers and newborns), caregiver behaviors (e.g., intention to use formula milk), and lack of an enabling environment for breastfeeding mitigate the expected effect of prolonged SSC on EBF3D, even when breastfeeding is initiated early.

The high proportion of mothers who intended to provide infant formula to their newborns in the first few days of life (52.5% brought infant formula to maternity facilities at birth, 35.4% bought infant formula at and nearby the hospital) is consistent with previous findings that social norms both within and outside of the health facility encourage artificial feeding and low self-efficacy toward breastfeeding (32). Intention to breastfeed may be influenced by a number of factors, including family and social norms, work status, availability of childcare, planned cesarean birth, aggressive marketing of commercial milk formula; and by a lack of effective breastfeeding counseling particularly during ANC (55–57). This study found that women with previous children were more likely to provide EIBF, suggesting that previous experience may play a role in establishing breastfeeding as a norm and for gaining confidence in breastfeeding techniques as has been reported in other studies (58, 59). The data showed that women with primary school education or less were less likely to practice EBF3D because these women feed the newborns both infant formula (i.e., a practice allowed by a non-supportive environment) and other fluids (i.e., as a traditional practice). This is a concerning finding given that children of mothers with lower educational attainment are often at higher risk of poor infant health and require additional support (33, 60, 61). We found that no other associations between breastfeeding and education or occupation, with prevalence of formula use similar across women of all education levels and occupations. These findings suggest that social norms discouraging intention to breastfeed have spread widely across geographical regions and socioeconomic groups (27, 61–63).

The finding that 35.4% of mothers bought infant formula at or nearby the birth hospital and 5.9% received a free infant formula sample during their hospital stay suggests that breastfeeding is not being adequately protected or supported in maternity facilities. The association between the availability of infant formula in hospital (brought or purchased) and provision of free samples with reduced likelihood of EBF3D is consistent with previous findings that commercial milk formula advertising, promotional materials and formula availability can limit breastfeeding (32, 64). Commercial milk formula industry representatives often use tactics that circumvent hospital regulations to promote formula milk to pregnant women and new mothers, including collecting contact information and promoting products like commercial milk formula for pregnant women that are not covered by the Code (65). These practices undermine breastfeeding and the provision of breastfeeding support by health workers during ANC and at the time of birth.

Further, the data also indicate that breastfeeding promotion and support provided by health workers at ANC and birth contacts is insufficient to influence breastfeeding practices. Although all mothers have access to ANC and 76.6% made at least four ANC visits, only 66.3% received breastfeeding advice by a health worker during an ANC visit; and breastfeeding counseling from a health worker was not significantly associated with breastfeeding practices, suggesting inadequate quality or frequency of counseling. This study is limited in not being able to grade the quality and frequency of advice received. Women in the sample preferred going to private clinics for ANC (possibly due to convenience) but giving birth at public hospitals (possibly due to perceived quality of service). In fact, the availability of breastfeeding support is likely to be lower, and Code violations higher in private clinics than public hospitals (38). Only 55.2% of mothers reported receiving breastfeeding counseling and support by a health worker around the time of birth at hospital and the receipt of such support was not associated with increased EBF3D, suggesting that quality is suboptimal. Previous studies in Vietnam have shown that effective breastfeeding counseling and support has a positive effect on early and exclusive breastfeeding (66), however, if health workers have insufficient time or skills, quality of support provided will be low and they may recommend the use of infant formula when feeding difficulties or concerns arise (60). The fact that the first-time mothers were less likely to practice EIBF suggests that maternal experience and confidence are important contributors to feeding practices at the time of birth and call for extra support from health staff.

To be effective, breastfeeding support should be predictable, scheduled, and include ongoing visits with trained health professionals including midwives, nurses, and doctors, or with trained volunteers (67). Support may be needed to tailor advice to specific cultural, geographic, or social settings (67). In Vietnam, although reproductive health practice guidelines include breastfeeding counseling (25), counseling and preventive health are not covered by health insurance (68, 69), which may reduce health worker motivation to provide these services. In addition, only a few mothers in our sample received breastfeeding advice and support from village health workers or nutrition collaborators, indicating that community-based support services need to be strengthened (66, 70).

Several actions could be taken to strengthen EIBF and EBF3D based on study findings, focusing on facility policies and environments before during and after birth to promote, support and enable early and exclusive breastfeeding practices.

First, policies and environments in hospitals must be changed to limit the availability of commercial milk formula and encourage successful breastfeeding (32, 71). Vietnam’s national legislation on the Code is moderately aligned with the International Code of Marketing of Breast Milk Substitutes (scored at 74 out of 100) (18). Based on the Code in Vietnam (19, 20), recommended breastfeeding practices and the Code compliance (Criteria E1.3) have been integrated as one of 83 criteria under the National Hospital Standards and Accreditation for both public and private hospitals (21). However, clinics unaffiliated with hospitals are not regulated under these standards (21) while private hospitals have low motivation to meet criteria E1.3. The study further indicates that many public hospitals are not meeting Code regulations. In the longer term, it is critical that monitoring and enforcement of the Code is strengthened across all facilities providing maternity services. As an immediate first step, maternity hospitals must be mandated by decree to exclude formula entirely from hospitals (including preventing it being brought in by patients and families), ban the distribution of free formula samples and make it impossible to purchase formula on hospital grounds or in shops nearby birthing facilities.

Second, the strong association between immediate and prolonged SSC and EIBF (and potentially also with EBF3D), indicates that improving this practice for all births should be a high priority. Study data show that only 49.3% of women receive immediate SSC and of these, only 11.6% receive uninterrupted SSC for the recommended 90 min. Vietnam has guidelines for the implementation of early essential newborn care for both vaginal (22) and cesarean births (23), which have been gradually introduced to maternity facilities nationwide. Early essential newborn care has been demonstrated to be associated with improved newborn outcomes and breastfeeding practices in Vietnam (24, 29, 52). Roll out has used a systematic approach that includes updating policies and protocols, clinical coaching of staff using adult learning principles, modifications to environments and birthing room supports and introduction of a data-driven quality improvement process, that has resulted in sustained improvements in practices around birth (72). Priority should be given to the introduction and expansion of this approach nationally using existing facilitators and proven methods.

Third, and related to the second point above, early newborn care practices at cesarean and vaginal births with episiotomy should be improved to encourage the principles of effective early essential newborn care. This includes non-separation of all clinically stable newborns, immediate and prolonged SSC, initiation of breastfeeding while in SSC with the mother and effective breastfeeding counseling and support, including recognition of feeding cues, position, and attachment. Introduction requires a collaborative approach between obstetrics, pediatric and anesthesia staff, a practiced system of actions with allocation of roles and changes to operating room environments. It has been successfully introduced in some hospitals in Vietnam, with clearly defined methods and protocols that should now be introduced more widely (23, 52).

Ongoing efforts to limit unnecessary cesarean births and the use of episiotomy must continue. We found that the prevalence of cesarean birth and episiotomy were high and associated with lower prevalence of EIBF and EBF3D. The prevalence of cesarean birth (44.6%) in our study was higher than the national rate of 34.4% (10) and much higher than the WHO’s recommended prevalence of between 10 and 15% (12, 13, 46). The high rate of episiotomy (46.1% of all births and 83.3% of vaginal births) suggests that the practice is routine, rather than restricted as recommended by WHO (73). Episiotomy is considered a harmful procedure that is associated with postpartum hemorrhage, postnatal hospitalization for more than 4 days, and third- or fourth-degree perineal tears (32, 55, 74). Limiting these harmful and unnecessary procedures should be a high priority at all levels, beginning with national guidelines and regulations; and integrating with efforts to improve respectful and evidence-based maternal care, including those promoted by early essential newborn care. Dealing with incentives to conduct procedures is an ongoing challenge (13, 15, 52).

Fourth, linked with the second and third points above, breastfeeding counseling and support at both ANC contacts and at the time of facility birth must be strengthened. Adequate support requires staff to have appropriate skills, time and a motivating environment that supports effective practices. Early essential newborn care includes a focus on this area, for the period around birth. Efforts to improve counseling and support have been demonstrated to be effective in many settings (67). This gap may require improved medical, midwifery and nursing pre- and in-service training, task-shifting in clinical settings and re-organization of care environments to provide adequate skills, time, and space for adequate counseling (75). It may also be helpful in Vietnam to include breastfeeding support in insurance reimbursement packages for ANC and childbirth; and to strengthen the skills of village health workers or nutrition collaborators with special focus on first time mothers and those with lower education. Mass and digital media campaigns may be useful to create social norms that are more supportive of breastfeeding.

### Limitations

This study has several strengths, including representative population-based sampling, use of validated standardized questionnaires (32, 41–44), and use of hand-held devices to reduce errors during data collection and entry. The data allow identification of interventions that should be prioritized to prevent substantial economic and health losses (75). Several limitations are noted including purposive selection of sampling areas which may limit national representativeness; use of immunization household listings which were assumed to be complete; and inclusion of migrant women in district sampling lists who may not represent the socio-economic or cultural characteristics of women in the sampled province. The impact of these potential sampling biases is believed to be minor. In-person data collection occurred during the COVID-19 pandemic, which made some women hesitant to participate in the interview (a non-response rate of 14.6% was noted). It is not possible to know whether this group differed significantly on socio-demographic or other characteristics, although the non-response rate was similar between all provinces.

Other limitations include recall bias (since women up to 11 months post birth were included) and reporting bias (respondents may have been influenced by the desire to report recommended breastfeeding practices and not report non-recommended practices such as formula use). However, validity and reliability for recalled breastfeeding data is reported as relatively high for survey data, which may have a recall period up to 24 months (42). Further, previous validation of the mothers’ recall of immediate newborn care practices has shown high levels of agreement between observed and reported measures of initiation of SSC and duration of uninterrupted SSC in the first 24–72 h after birth (76). Although the validity and reliability of reported durations of SSC contact for recall periods of up to 11 months postpartum were not yet documented, mothers tend to remember well practices and events around birth even after 24 months (42). In addition, the relationships between EIBF and duration of SSC and associations of EBF3D with availability of formula were consistent between all provinces and different socio-economic groups suggesting that systematic bias was not a problem. Finally, this analysis of existing survey data meant researchers were unable to measure all potential factors associated with the likelihood of breastfeeding, including previous breastfeeding experiences, body mass, smoking, birth companion, difficulties initiating breastfeeding, availability of childcare or child support, and perinatal depression. Similarly, the quality and frequency of breastfeeding counseling and advice received could not be determined.

## Conclusion

This study of 726 mothers with infants aged 0–11 months from provinces representative of the socio-economic characteristics of Vietnam found a low prevalence of EIBF (49.7%) and EBF3D (18.0%), and high prevalence of infant formula use (79.3%). Barriers to recommended breastfeeding practices in the first days of life included provider behaviors and medical procedures (cesarean birth, episiotomy, lack of immediate and uninterrupted SSC and limited effectiveness of breastfeeding counseling and support during antenatal care and around the time of birth) and unsupportive breastfeeding environments (bringing or purchasing formula milk at the health facility and receipt of free infant formula samples) at maternity facilities.

To improve breastfeeding practices, both health care provider practices and environments at maternity facilities must be improved. Quality improvement approaches to strengthen staff coaching, protocols, and health facility environments should be scaled up to ensure consistent implementation of early essential newborn care, including immediate and prolonged SSC, and to reduce unnecessary cesarean sections and episiotomies. In tandem, action is urgently needed to improve breastfeeding counseling and support at all facility and community contacts around birth; mass and digital media campaigns may be useful to create social norms that are more supportive of breastfeeding from birth and during the first days of life. Stronger enforcement of national policies to regulate the presence of commercial milk formula industry representatives, provision of free samples, and availability of infant formula in public and private health facilities is needed, including ensuring that formula cannot be purchased in or around hospitals.

## Data availability statement

The raw data supporting the conclusions of this article will be made available by the authors, without undue reservation.

## Ethics statement

This study was conducted according to the guidelines of the Declaration of Helsinki and approved by the Institutional Review Board (or Ethics Committee) of FHI 360 (protocol code 1383644; approved on April 16, 2019) and Hanoi University of Public Health (protocol code 019-501/DD-YTCC; approved on June 12, 2019). Written informed consent for participation was not required for this study in accordance with the national legislation and the institutional requirements.

## Author contributions

TN, JC, JM, and RM: conceptualization. TN, JC, and JM: methodology, validation, data curation, and writing—original draft preparation. TN: software, formal analysis, investigation, visualization, supervision, and project administration. RM: resources and funding acquisition. TN, JC, HTT, TAH, AW, RM, and JM: writing—review and editing. All authors have read and agreed to the published version of the manuscript.
