# Supplementary material for: Birth and newborn care policies and practices limit breastfeeding at maternity facilities in Vietnam
Source: Front Nutr. 2022 Oct 28;9:1041065. doi: 10.3389/fnut.2022.1041065 (PMC9668009; doi:10.3389/fnut.2022.1041065)
Supplement: Supplementary file 1 [file Data_Sheet_1.PDF]

TOOL 8.2. QUESTIONNAIRE FOR MOTHERS WITH CHILDREN UNDER 12 MONTHS

1. BACKGROUND AND CHARACTERISTICS

No	Question	Code	variable name
1	Interviewer's name	Select name	int
2	Province/ city that the interview is being conducted in:	1= HCM city<br>2= Binh Duong<br>3= Bac Ninh	prov
3	PSU code	Enter code 1-30	psu
4	What is your name/code of mother?	....	mname
5	Enter women's code	***2 digits***	mcode
6	Unique ID (automatically generated)	***Provivnce.PSU.Women.Type***<br>***(check with code on the spread sheet)***	id
7	What is your ethnicity?	Kinh .... 1<br>Other (specify)………………………….. 77	ethnic
8	What is your year of birth?	____________	myob
9	What is your marital status?	Unmarried .... 1<br>Married .... 2<br>Widowed/Divorced/Separated .... 3<br>Other (specify) 77	marital
10	Are you living with your husband/partner?	1. Yes<br>0. No	plivewt
11	What is the highest level of education you have **completed**?	Never attended school ....0<br>Primary school ....1<br>Junior secondary school ....2<br>Secondary school.......................................3<br>Diploma...4<br>Bachelors/Masters/higher……………...5	medu
12	How many biological children do you have?	☐ children	bchild
13	How many biological children under 5 years of age do you have?	☐ children	child
14	How many biological children under 12 months do you have?	☐ children	under12
15	What is the name of youngest child (the one most recently born)?	………………………………	ycname
16	Is the child a boy or girl?	Boy…………………………………..1<br>Girl…………………………………..2	ycsex
17	What is the date of birth of this child?	____/ _____ /______<br>Date / Month / Year<br>Identify the child belongs to group 2 or group 3?<br>1. < 6 months<br>2. 6-11.9 months	ycdob<br><br>group

18	What was your occupation prior giving birth to (NAME)?	Farmer (planting, feeding animals, farming)/fisherfolk .... 1<br>Green-collar: worker, technician, janitor .. 2<br>White-collar: educated staff, office clerk, doctor, teacher, enterprise owner .... 3<br>Small trader/ self-employed / small self-owned business/services (tailor/hairdresser/builder)/freelancer........ 4<br>Unemployed / Homemaker/ student… ..... 5<br>Other (specify)……………………………77	mjob
19	Before giving birth to (NAME), did you contribute to the Social Security Fund?<br>*This is the money taken from your paycheck. Then the fund provides some money to you during sickness, maternity, labor accident, old age…*	Yes .... 1<br>No .... 0<br>Don't know .... 98<br>No Response.. 99	insur
20	Have you gone back to work after giving birth to your child (e.g., to the field, previous employment)?	Yes .... 1 -> (skip Q24)<br>No …………0 → **Move on Q24**	**backwork**
21	How long after giving birth to (NAME) did you go back to work?	……….. month(s)……….. days	backdate
22	In the last 7 days, how many days did you work outside the home?	☐ Days	daywork
23	On days you work outside the home, for how many hours are you out on average?	☐☐ Hours	hourwork
24	Why haven't you returned to work since giving birth?<br><br>***Multiple responses possible.***	1. Still on maternity leave<br>2. Lost my previous job and I'm looking for another one<br>3. Waiting to hear if I got a new job to which I applied<br>4. No one to take care of my children or childcare is too expensive<br>5. I could not find work, lack of business<br>6. No suitable work available in the area or relevant to my skills, capacities<br>7. I prefer to stay home<br>8. My health is not good enough<br>9. The health of my child is not good enough<br>77. Other (specify)_____________________<br>99. Do not know<br>88. Response refused	whynot
25	Could you please name all the people that (NAME) has lived in the same house with for at least 15 days of the previous 30 days??<br><br>***Multiple responses possible.***<br>***Probe:*** Anyone else?	With me (the mother) .... 1<br>My spouse, partner .... 2<br>Elder siblings.. 3<br>Grandparents .... 4<br>Other relative.. 5<br>Nanny (hired child caregiver)...................... 6<br>Other (specify)………………………..…..77	hhmem
26	Who looks after (NAME) daily?<br><br>***Multiple responses possible.***<br>***Probe:*** Anyone else?	Me (the mother).. 1<br>My spouse, partner .... 2<br>Siblings... 3<br>Grandparents 4	caregiver

		Other relative ....5<br>Nanny (hired child caregiver)......................6<br>Teachers (in childcare center) ....7<br>Other (specify)……………………………77	
27	What is your husband/partners' current main occupation?	Farmer (planting, feeding animals, farming)/fisherfolk .... 1<br>Green-collar: worker, technician, janitor .. 2<br>White-collar: educated staff, office clerk, doctor, teacher, enterprise owner .... 3<br>Small trader/ self-employed / small self-owned business/services (tailor/hairdresser/builder)/freelancer........ 4<br>Unemployed / Homemaker/ student… .... 5<br>Other (specify)……………………………77	pjob
28	When applying for a job, have you ever been required to **have a pregnancy test** or **doctor's diagnostic certificate** to confirm that you are not pregnant?	1. Yes.<br>0. No<br>***2.*** Have not applied for a job	pregtest
29	When applying for a job, have you ever **been asked about your plans to have a baby** or **your marital status**?	1. Yes.<br>0. No<br>***2.*** Have not applied for a job	famask

2. ANTENATAL CARE, FEEDING PRACTICES AND CHILD HEALTH

Now I would like to ask you some questions about pregnancy, delivery and breastfeeding.

No	Question	Code	variable name
3	Did you receive antenatal care or an ultrasound during your pregnancy with (NAME)	Yes .... 1<br>No .... 0 → **Move on Q11**	anc
4	Where did you go for antenatal care or ultrasound?<br><br>***Multiple responses possible***	Public hospital (provincial and central levels) .... 1<br>Public polyclinic, district health center... 2<br>Private/International hospital/clinic .... 3<br>Commune health center .... 4<br>At home…………………………………5<br>Other (specify)…………………………..77	ancplace
5	How many times did you receive antenatal care during this pregnancy that included the following?<br><br>*(ANC: Lying on a bed or obstetric examination table, health worker measuring blood pressure, waist circumference; checking the uterus, fetal heart rate, the appendages and asking the health situation. In some cases, having more tests)*<br>***Probe*** *to identify the number of times antenatal care was received. If a range is given, record the minimum number of times antenatal care received.*<br><br>(Note: If you get both ANC and ultrasound in one visit, count 1)	Number of times................................. __ __<br>Don't know.. 98	anctime

6	Of the <mentioned the number in question 4> times you received antenatal care, **how many times was it paid mainly by <read each option>?**<br><br>***Multiple responses possible***<br>***Probe:*** Anyone else?<br>*(Note: the add-up total must equal to the total in Q4)*	Health insurance …… 1 __ __ times<br>Company/private health insurance 3 __ __ times<br>Out of pocket (e.g., Myself)……2 __ __ times<br>Other (specify)…. 77 __ __ times	ancpay1<br>ancpay2<br>ancpay3<br>ancpay4
7	To go for antenatal care, how many times did you **<read each option>**?	Take a break during working hours, with salary __ __ times<br>Take a break during working hours, without salary __ __ times<br>Have checkup out of working hours: ……………… __ __ times	offpay<br>notpay<br>offwork
8	What is the total number of times you received ultrasound ONLY during your pregnancy?	__ __ times	ulttot
11	When you were **pregnant** with (NAME), did you receive any advice about breastfeeding from **anyone**?<br><br>***Multiple responses possible***	Health workers from a health facility ..... 1<br>Hamlet health worker .... 2<br>Nutrition collaborator .... 3<br>Women Union staff .... 4<br>Husband .... 5<br>Mother/Mother in law............................ 6<br>Other Family members .... 7<br>Neighbors/ Friends/co-workers .... 8<br>Other (*specify*) .... 77<br>No one………………..…………………9	ancadv
12	When you were **pregnant** with (NAME), did you receive any advice about breastfeeding from **any other sources**?<br><br>***Multiple responses possible***	Television……………………………...1<br>Internet (social media, search engine [e.g., Google])…………..2<br>Loudspeaker/Radio………………………3<br>Books/newspapers/magazines/leaflets…....4<br>Advertisement boards, posters……………5<br>Prenatal courses…………………………..6<br>Other events (specify)……………………77<br>Nowhere.. ..9	ancinfo
13	During your pregnancy with (NAME), did you drink milk for pregnant women?	1. Yes<br>0. No	pregmilk
14	During your pregnancy with (NAME), did you take iron folate or micronutrient supplement?	1. Yes<br>0. No	suppl
15	Where did you give birth to (NAME)?	Public hospital (provincial and central levels) .... 1<br>Public polyclinic, district health center... 2<br>Private/International hospital/clinic .... 3<br>Commune health center .... 4<br>At home…………………………………5<br>Other (specify)…………………………..77	bplace
16	Did you have a cesarean delivery when you gave birth to (NAME)?	Yes……………1<br>No...................... 0 → **Move on Q18**	cesarean
17	Why did you have cesarean delivery this time?	Previous cesarean delivery.............................. 1	cswhy

	Multiple responses possible	Indicated by a doctor due to medical reasons from mother or the child .... 2<br>Thought that cesarean delivery is safer............ 3<br>Ability to select "good" hour and date of birth 4<br>Convenience (e.g., at the shift of doctor with used for ANC) .... 5<br>Avoiding pain... 6<br>Suggestion from family members, friends....... 7<br>Fear of later effect of vaginal delivery to sexual life, lower pelvic trauma etc............................ 8<br>Other (specify) .... 77<br>Don't answer 99	
18	Did you have an episiotomy when you gave birth to (NAME)?<br>*(An episiotomy, also known as perineotomy, is a surgical incision of the perineum and the posterior vaginal wall generally done by a midwife or obstetrician).*	Yes .... 1<br>No 0	epitomy
19	After giving birth, was (NAME) placed on your chest skin to skin?	Yes .... 1<br>No …………….0 → **Move on Q23**	s2s
20	How long did it take from (NAME)'s first cry until (he/she) was put onto your chest?	Immediately or <1 minute .... 1<br>1-10 minutes .... 2<br>11-59 minutes .... 3<br>≥ 60 minutes…………………………… 4	s2sdur
21	For how long was (NAME) kept skin to skin on your chest with you with no break or separation?<br><br>*(if the mother answers a range, use the upper value)*	<10 minutes……………………………...1<br>10–29 minutes…………………………...2<br>30–59 minutes…………………………...3<br>60–89 minutes…………………………...4<br>≥ 90 minutes……………………………..5	s2slong
22	Did (NAME) receive its first breastfeed before s/he was separated from you?	Yes .... 1<br>No 0	s2sbrf
23	How long after birth, did (NAME) stay in the same room with you (rooming in)?	After birth……………..…………………1<br><1 hour..…………………………………2<br>1- 5 hours.………………………………..3<br>6 -24 hours.………………………………4<br>Sau 24 hours.…………………………….5	withmom
24	What was the weight of (NAME) at birth?	_ _ _ _ grams	bwght
25	How many gestational weeks were you when you gave birth to (NAME)?	_ _ gestation weeks	gestage2
26	How many days after giving birth to (NAME) did you stay in the health facility?	_ _ days	bstay
27	Have you ever fed (NAME) with your own milk?<br>(took milk from you from the breast or another way such as by spoon, cup or bottle)	Yes……………………………………1-> ask question 28<br>No……………………0 → Ask questions 27b, **Q29, skip questions 41-46**	bmilk

27b	What are the reasons for not breastfeed the child?	Mother have diseases or on medications that did not allow to breastfeed …………….1<br>Mother-child separation (could also due to disease of the child)……….. ….2<br>Khác………………………..77 (Ghi rõ………)	
28	How soon after birth did you put (NAME) to the breast for the first time?<br><br>If the mother answers "immediately", interviewers ask the mother again about the exact time and record the appropriate time.<br>*If less than 1 hour, circle 1.*<br>*If less than 24 hours, circle 2 and record hours.*<br>*If more than 24 hours, circle 3 and record days.*	Within 1 hour……………………………1<br>Number of hours………………………..2<br>Number of days…………………………3<br>[ ][ ] . [ ] Hours [ ][ ] Days	firstmilk
29	Has (NAME) ever been fed breastmilk from another woman?	Yes, directly from other women (from the breast or expressed milk) ..……..1<br>Yes, from a human milk bank………………2<br>No…………………………………………0	usedm
30	Within the first 3 days after birth, did anyone show you how to breastfeed?	Yes……………………………………....1<br>No……………..2 → **Move on Q32**	adv3
31	Who showed you how to breastfeed?<br><br>***Multiple responses possible.***<br>***Probe:*** Anyone else?	Health workers from a health facility ..... 1<br>Hamlet health worker .... 2<br>Nutrition collaborator .... 3<br>Women Union staff .... 4<br>Husband... 5<br>Mother/Mother in law............................ 6<br>Other Family members .... 7<br>Neighbors/ Friends/co-workers .... 8<br>Other (*specify*) .... 77<br>Nobody…………………..…………………9	advwho
32	In the first 3 days after birth, was (NAME) given any <read each response>?	Breastmilk………………………………1 0<br>Breastmilk from another woman…………..1 0<br>Infant Formula /other infant milk……….1 0<br>Plain water .... 1 0<br>Sugar or glucose water .... 1 0<br>Honey .... 1 0<br>Lemon juice/ herbal tea (e.g., licorice root) 1 0<br>Anything else? (specify)......................... 1 0<br>Nothing besides breastmilk.................... 1 0	otherfood
33	Did you or your family member <u>bring</u> any infant formula to the health facility when you gave birth to (NAME)?	Yes .... 1<br>No ……………………………………… 0	forbring
34	Did you or your family member <u>purchase</u> any infant formula at or near the health facility shortly after you gave birth to (NAME)?	Yes .... 1<br>No ……………………………………….0	forbuy
35	Were you given infant formula for <u>**FREE**</u> while in the health facility?	Yes .... 1<br>No ……………………………………… 0	freesamp
36	During your stay at the hospital, did a representative from a milk company <u>approach</u> you to give you information or advice about formula, bottles or pacifiers?	Yes……………………………………1<br>No 0	prohosp

37	During your stay at the hospital, did a representative from a milk company <u>approach</u> you to ask for your personal information such as your address, phone number, email address, Facebook, Zalo…?	Yes……………………………………….1<br>No 0	prohosp2
38	Within 1 month after discharge, did a representative from a milk company call, send email, message to share information about infant formula, bottles, or pacifiers?	Yes……………………………………… 1<br>No 0	prohome
39	Within 1 month after discharge, did a representative from a milk company meet you in person to share information about infant formula, bottles, or pacifiers?	Yes……………………………………… 1<br>No 0	prohome2
40	While at the hospital as well as after you returned home, did relatives, friends, or colleagues give you infant formula as a gift?	Yes…………………………………… 1<br>No……………………………………. 0	fgift
41	Do you currently breastfeed (NAME)?	Yes....................... 1 → **Move on Q44**<br>No................. 0	stillbf
42	At what age (in months) was (NAME) weaned?<br>(If the mother answered a range, encourage her to give the best estimate)	………..months………..days<br><br>Never breastfeed…..…..0	stopbf
43	Why did you weaned (NAME) then?<br><br>***Multiple responses possible***<br>After each choice, bonus asking: Is there any other reason?	I was sick.. 1<br>Problem with breast (pain, cracked nipples, engorgement) .... 2<br>I didn't have enough time to feed .... 3<br>I felt that breastmilk was not enough......... 4<br>I felt that my breastmilk was not good ......5<br>I got pregnant / had another baby.............. 6<br>I went back to work .... 7<br><br>My child refused/lazy to eat...................... 8<br>My child stopped breastfeeding him/herself (e.g., sick or any other reasons).......................... 9<br>My child grew up already ....10<br>Other (specify) 77	whystop
44	Did you **ever** have any difficulties breastfeeding (NAME)?	No difficulty …… 0 → **Move on 47**<br>There are difficulties …………… .1	diff
44	What difficulties do you have?<br><br>***Multiple responses possible***<br>After each choice, PVV asks: Is there any other difficulties?	Infant resisted to breastfeed ....1<br>Infant did not suck well/did not attach well .... 2<br>Infant was choking, vomiting, refluxing ...3<br>Infant was sick, teething or got thrush ......4<br>I didn't have enough milk (says/thinks) ... 5<br>I had problems with breast (pain, crack nipples, engorgement) .... 6<br>I had pain associated with episiotomy or C-Section…………………………………...7	diffwhat

		Not enough time to breastfeed, had to go to work .... 8<br>I was sick and unable to breastfeed............9<br>I did not know how to and was not confident with breastfeeding ....10<br>My parents or husband urged me to give infant formula or other food to my baby ....11<br>Other (Specify)...77	
45	Did you seek help or advice from anyone for these difficulties?	Yes 1<br>No 0 → **Move on Q47**	diffadv
46	Who gave you help or advice?<br><br>***Multiple responses possible***<br>After each choice, PVV asks: Is there anyone else?	Health workers from a health facility ..... 1<br>Hamlet health worker............................. 2<br>Nutrition collaborator............................. 3<br>Women Union staff................................ 4<br>Husband .... 5<br>Mother/Mother in law .... 6<br>Other Family members........................... 7<br>Neighbors/ Friends/co-workers.............. 8<br>Other (*specify*).. 77<br>Nobody…………………..…………………9	diffwho

Now I would like to ask you about all other liquids that (NAME) may have had yesterday during the day or the night. Please include liquids consumed outside of your home.
Did (NAME) drink (name of item) yesterday during the day or the night: (Variable name Q47: **feed24h)**

No	Question	Code	Yes	No	Don't know
47	Think about **<u>the time from when (NAME) woke up yesterday morning to when (NAME) woke up this morning,</u>** was (NAME) allowed to feed/ drink any of the following...<br>***(interviewer read each option)?***	Breastmilk	1	2	98
		Infant Formula /other infant milk	1	2	98
		Liquid milk formula	1	2	98
		Other milk (example: Packed fresh milk, fresh milk, sweetened condensed milk)	1	2	98
		Products from milk (example: Cheese, yogurt, cakes ...)	1	2	98
		Plain water, herbal water	1	2	98
		Canned fruit juice / sugar / herbal juice	1	2	98
		Broth / rice broth / congee juice	1	2	98
		Fruit (including crushing / pressing juice)	1	2	98
		Soft / thick / thick foods (e.g., dough, porridge) cook or eat right away	1	2	98
		Rice, noodles, instant noodles, potatoes, cassava	1	2	98
		Vegetables	1	2	98
		Meat, fish, shrimp, eggs	1	2	98
		Candy, cake, snack or other junk food	1	2	98
		Vitamins, minerals, syrup	1	2	98
		Other (specify……………………..……..)	1	2	98

48	**<u>From when (NAME) woke up yesterday morning to when (NAME) woke up this morning,</u>** did (NAME) drink anything from a bottle with a teat?	Yes ....1<br>No 0	ateat

Now I would like to ask you about any illness (NAME) had in the past two weeks.

No	Question	Code	
49	Was (NAME) ill in the past two weeks?	Yes .... 1<br>No …………0 → **Move on Section 3**	sick
50	If yes, what were his/her main symptoms?<br><br>***Multiple responses possible.***	Fever ....1<br>Cough/Cold/Fast breathing/shortness of breath ....2<br>Diarrhea...3<br>Other (specify) 77	sickwhat
51	For the illness, was (NAME) taken for a checkup? Where?<br><br>***Multiple responses possible***	Public hospital (provincial and central levels) .... 1<br>Public polyclinic, district health center... 2<br>Private/International hospital/clinic .... 3<br>Commune health center .... 4<br>At home for self-treatment……………….5<br>At home for naturally recover……………6<br>Other (specify)…………………………..77	sickwhere<br><br>*If = 5, 6 -> Part 3*
52	Who brought (NAME) for the checkup?<br><br>***Multiple responses possible***	Me (the mother) .... 1<br>My spouse, partner...................................... 2<br>Elder siblings .... 3<br>Grandparents .... 4<br>Other relative (aunt, uncle) .... 5<br>Nanny (hired child caregiver) .... 6<br>Teachers (in childcare center) .... 7<br>Other (specify)…………………………….77	sickwho

3. DETERMINANTS

3.1. (Intention)

Now I want you to think ahead to a time when you might have another child. For these next statements, please respond for the actions you would take <u>if you had another child</u>. Please tell me whether you strongly disagree, disagree, disagree somewhat, agree somewhat, agree or strongly agree with each of the following statements.

If the answer is "don't know," code as 98.

1	2	3	4	5	6
**Strongly disagree**	**Disagree**	**Somewhat Disagree**	**Somewhat Agree**	**Agree**	**Strongly agree**

No	**Question**<br>***Please provide your opinion on the following statements:***<br>***('I' here means talking about you and not talking about the person interviewing you)***	Code
1	The next time I get pregnant, I will drink milk for pregnant women	plan1
2	The next time I have a baby, I will breastfeed him/her within 1 hour after giving birth	plan2
3	The next time I have a baby, I will not give him/her anything other than breastmilk in the first 3 days after birth	plan3
4	The next time I have a baby, I will not give him/her any infant formula for the first 6 months.	plan4

3.2. **(Knowledge of breastfeeding)** Now I would like to ask your opinion about infant feeding.

No	Question	Code	
1	How long after birth should a newborn start breastfeeding?<br><br>If the mother answers "immediately", interviewers ask the mother again about the exact time and record the appropriate time.<br>*If less than 1 hour, circle 1.*<br>*If less than 24 hours, circle 2 and record hours.*<br>*If more than 24 hours, circle 3 and record days.*	Within 1 hour .... 1<br>Number of hours .... 2<br>Number of days .... 3<br>[ ][ ] Hours [ ][ ] Days<br><br>Don't know 98	kfm<br>kfmh<br>kfmd
2	If a mother thinks her **4-month-old** infant is not getting enough breastmilk, what should she do?<br><br>***Multiple responses possible.***<br><br>***Probe once:*** Anything else?	Breastfeed more often/more frequently .... 1<br>Give infant formula .... 2<br>Give infant other liquids/foods (e.g. water/fruits juice /Rice porridge/ rice flour).......... 3<br>Mother needs to drink more water .... 4<br>Mother needs to eat more food .... 5<br>Mother needs to eat special food .... 6<br>Consult with health care workers (doctor, nurse, midwife, nutrition collaborator/village health worker) .... 7<br>Seek opinions from social media networks .... 6<br>Other (Specify): .... 77<br>Don't know 98	klackm
3	Until what month should a mother give her infant <u>**ONLY**</u> breastmilk and <u>**NO**</u> other food, water or infant formula?	[ ][ ] Months<br><br>Don't know..........98<br>No response..........99	kebf
4	Until what month should a mother continue to breastfeed?<br><br>***Write down the age as months.***	[ ][ ] Months<br>Don't know ....98<br>No response..........99	kcbf

3.3. (Beliefs)

I would like to ask your opinion about some other feeding practices. Please tell me whether you strongly disagree, disagree, disagree somewhat, agree somewhat, agree or strongly agree with each of the following statements.

If the answer is "don't know," code as 98.

1	2	3	4	5	6
**Strongly disagree**	**Disagree**	**Somewhat Disagree**	**Somewhat Agree**	**Agree**	**Strongly agree**

No	Question	Variable name
	Please tell me your opinion about the following statements.<br>***"I" in all of the following statements refers to the respondent and not the interviewer.***	
1	If I **breastfeed** my infant **within 1 hour after giving birth**, it'll be good for my child's health	belief1
2	If I am breastfeeding, but <u>**DO NOT**</u> give my newborn infant formula during the first 24 hours after birth, s/he will be hungry.	belief2
3	If I feed my infant <u>**ONLY**</u> breastmilk and <u>**NO**</u> other food, water or infant formula, until s/he completes 6 months, I am giving my infant all the nutrients s/he needs to be healthy.	belief3
4	If I feed my infant a combination of breastmilk and infant formula until s/he completes 6 months, I am giving him/her the <u>**BEST**</u> possible nutrition.	belief4

5	After I start to give infant formula to my infant, my body will produce less breastmilk.	belief5
6	If I continue breastfeeding until my infant completes 2 years, it is good for my infant's health.	belief6
7	If I drink milk for pregnant women, my newborn will be smart and healthy.	belief7
8	I would feel uncomfortable to breastfeed my infant in a public place.	belief8

3.4. (Social Norms)
I would like to ask your opinion about some social norms of other feeding practices. Please tell me whether you **strongly disagree, disagree, disagree somewhat, agree somewhat, agree or strongly agree** with each of the following statements.
If the answer is "don't know," code as 98.

1	2	3	4	5	6
**Strongly disagree**	**Disagree**	**Somewhat Disagree**	**Somewhat Agree**	**Agree**	**Strongly agree**

No	Question	Variable name
1	**Most people who are important to me (e.g. family members, friends…)** think that an infant needs formula milk in the first week after birth.	sn1
2	Most people who are important to me (e.g. family members, friends…) would approve of my giving my baby infant formula before she/he reaches 6 months of age.	sn2
3	Most people who are important to me (e.g. family members, friends…) would approve of me breastfeeding my baby in public places.	sn3
4	**Most of the pregnant women I know** consume(d) milk for pregnant women.	sn4
5	**Most of the women with an infant I know** fed their infant breastmilk within 1 hour after vaginal delivery.	sn5
6	Most of the women with an infant I know fed their infant breastmilk within 1 hour after caesarean delivery.	sn6
7	Most of the women with an infant I know fed their infant formula milk in the 1 first week of the infant's life.	sn7
8	Most of the women with an infant I know fed their infant only breastmilk, and no other food, water, or infant formula for the first 6 months of the infant's life.	sn8
9	Most of the women with an infant I know breastfed their infant in a public place.	sn9
10	Most of the women with an infant I know feed their infant milk formula when they take them out of the house and need to feed them.	sn10

3.5. (Self-Efficacy)
I would like to ask your opinion about some other feeding practices. Please tell me whether you are **very unconfident, unconfident, somewhat unconfident, somewhat confident, confident, or very confident** in response to the statement.
As you read the responses, point to each box.
If the respondent does not know, code 98.

1	2	3	4	5	6
**Very unconfident**	**Unconfident**	**Somewhat unconfident**	**Somewhat confident**	**Confident**	**Very confident**

No	Question	Code
1	My body can produce enough colostrum to feed my newborn within one hour after birth.	conf1
2	My breastmilk is of good enough quality to nourish my infant so that my infant does not need any other food, water, or infant formula until s/he has completed 6 months.	conf2

3	If I go back to work before my infant is six months old, I will have to start feeding him infant formula or semi-solid/solid foods.	conf3
4	I can continue to breastfeed my infant until she/he **reaches 24 months of age**.	conf4
5	I can breastfeed in a public place if my infant is hungry.	conf5
7	If money were not an issue, I would stay home to feed my infant during his/her first 6 months.	conf7
8	If money were not an issue and I could stay at home for the first 6 months, I would only breastfeed my infant and wouldn't feed him/her with any other food or drink or formula milk during the first 6 months.	conf8
9	When I look at the formula label in a store or supermarket, I will recognize which age range the formula is for.	conf9
10	If a representative from a milk company recommends a new infant formula, I will buy it for my infant.	conf10
11	If a health worker recommends a new infant formula, I will buy it for my infant.	conf11
12	If a friend of mine recommends a new infant formula, I will buy it for my infant.	conf12
13	If members of my social network group(s) recommend a new infant formula, I will buy it for my infant.	conf13

4. MEDIA EXPOSURE AND SOCIAL GROUP AND EVENT PARTICIPATION

Now I would like to ask you some questions about media exposure

No	Question	Code	Variable name
1	During last 30 days, did you see any information **on breastfeeding** on television, screens at a health care center, supermarkets, elevators or outdoors?	Yes .... 1<br>No ………………0 → **Move on Q3**<br>Don't know/don't remember.................. 98	media1
2	In the past 30 days, how often did you see the information on breastfeeding from any of the above media? Was it…<br>***Use Show card***	Daily (7 days/week) .... 1<br>Several times a week (2-6 days/week) .... 2<br>About once a week .... 3<br>Less than once a week (≤ 3 days/month)............ 4<br>Don't know/don't remember.................. 98	media2
3	<u>**Now, I am asking about milk formula advertisements**</u>. During the last 30 days, did you see any information on <u>**milk formula**</u> on television, screens at supermarkets, elevators or outdoors?	Yes .... 1<br>No …………….. 0 → **Move on Q5**<br>Don't know/don't remember.................. 98	media3
4	In the past 30 days, how often did you see information on <u>**milk formula**</u> on television, screens at supermarkets, elevators or outdoors? Was it…<br>***Use show card***	Daily (7 days/week) .... 1<br>Several times a week (2-6 days/week) .... 2<br>About once a week .... 3<br>Less than once a week (≤ 3 days/month)............ 4<br>Don't know/don't remember.................. 98	media4
5	In the past 6 months, have you been a member of any **online social group** for mothers and other caregivers of infants and young children, such as infant clubs or parenting groups?	Yes ....1<br>No ....0<br>Don't know/don't remember.................. 98	media5
6	In the past 6 months, have you participated in any **online events or activities** hosted for mothers and other caregivers of infants and young	Yes ....1<br>No ....0<br>Don't know/don't remember.................. 98	media6

	children, such as photo contests or promotional sales on e-commerce platforms?		
7	In the past 6 months, have you been a member of any **off-line social groups** for mothers and other caregivers of infants and young children, such as infant club or parenting group?	Yes ....1<br>No ....0<br>Don't know/don't remember 98	media7
8	In the past 6 months, have you attended **any classes** on parenting or infant and young child feeding?	Yes ....1<br>No ....0<br>Don't know/don't remember 98	media8
9	In the past 6 months, have you attended any **events or activities** hosted for mothers and other caregivers of infants and young children, such as infant fairs/ festivals?	Yes ....1<br>No ....0<br>Don't know/don't remember 98	media9

5. BREASTMILK SUBSTITUTE PROMOTION
(Questions are multiple-choice)

No	Question	Code	variable name
1	In the past 30 days, did you receive **<read choice one by one>** of Milk for pregnant women?	Advice or recommendation ....1<br>Free sample ....2<br>Coupons ....3<br>Gift ....4<br>Did not receive 5	assess1<br><br><br><br>chọn 5-> q4
2	**From whom** did you receive <list what indicated in questions 1>?	Health workers in public health facilities......... 1<br>Health workers in private health facilities........ 2<br>Village health workers, nutrition / population collaborators, women union representatives .... 3<br>Partner/relative/friend .... 4<br>Shop/pharmacy personnel .... 5<br>Representative of a BMS company.................. 6<br>Can't remember.. 98<br>Other (Specify)..77	alwho1
3	**Where did** you receive <list what indicated in questions 1-4 >**?**	Public health facilities...................................... 1<br>Private health facilities..................................... 2<br>Home.. 3<br>Shops/ Pharmacies .... 4<br>Onsite events, conferences .... 5<br><br>Television, Radio, Magazine, newspapers........ 6<br>Poster, Billboard .... 7<br><br>Social media (e.g. Facebook, Instagram, mobile chat apps) .... 8<br>Internet (e.g., online shops of a BMS company or food company, and of health workers) .... 9<br>Online events, conferences .... 10<br><br>Can't remember .... 98<br>Other (Specify)..77	alwhere1
4	In the past 30 days, did you receive **<read choice one by one>** of Formula	Advice or recommendation ....1<br>Free sample 2	assess2

	milk for infants and toddlers (0+ months)?	Coupons ....3<br>Gift ....4<br>Did not receive 5	 <br>chọn 5-> q7
5	**From whom** did you receive <list what indicated in questions 1>?	Health workers in public health facilities......... 1<br>Health workers in private health facilities........ 2<br>Village health workers, nutrition / population collaborators, women union representatives .... 3<br>Partner/relative/friend .... 4<br>Shop/pharmacy personnel .... 5<br>Representative of a BMS company.................. 6<br>Can't remember... 98<br>Other (Specify)...77	alwho2
6	**Where did** you receive <list what indicated in questions 1-4 >**?**	Public health facilities...................................... 1<br>Private health facilities..................................... 2<br>Home.. 3<br>Shops/ Pharmacies .... 4<br>Onsite events, conferences .... 5<br><br>Television, Radio, Magazine, newspapers........ 6<br>Poster, Billboard .... 7<br><br>Social media (e.g. Facebook, Instagram, mobile chat apps) .... 8<br>Internet (e.g., online shops of a BMS company or food company, and of health workers) .... 9<br>Online events, conferences .... 10<br><br>Can't remember .... 98<br>Other (Specify)...77	alwhere2
7	In the past 30 days, did you receive **<read choice one by one>** of Bottle and artificial teats?	Advice or recommendation............................1<br>Free sample ....2<br>Coupons ....3<br>Gift ....4<br>Did not receive 5	assess4<br><br><br><br>chọn 5-> part 6
8	**From whom** did you receive <list what indicated in questions 1>?	Health workers in public health facilities......... 1<br>Health workers in private health facilities........ 2<br>Village health workers, nutrition / population collaborators, women union representatives .... 3<br>Partner/relative/friend .... 4<br>Shop/pharmacy personnel .... 5<br>Representative of a BMS company.................. 6<br>Can't remember... 98<br>Other (Specify)...77	alwho4
9	**Where did** you receive <list what indicated in questions 1-4 >**?**	Public health facilities...................................... 1<br>Private health facilities..................................... 2<br>Home.. 3<br>Shops/ Pharmacies .... 4<br>Onsite events, conferences .... 5<br><br>Television, Radio, Magazine, newspapers........ 6<br>Poster, Billboard .... 7<br><br>Social media (e.g. Facebook, Instagram, mobile chat apps) 8	alwhere4

		Internet (e.g., online shops of a BMS company or food company, and of health workers) .... 9<br>Online events, conferences .... 10<br><br>Can't remember .... 98<br>Other (Specify)..77	

6. MATERNITY PROTECTION (ONLY ASK FOR EMPLOYED WOMEN)

(Only for women with a child who were in paid or unpaid work before birth, namely codes 2, 3 in Q 18-section 1)
Now, I would like to ask about maternity leave benefits that you received or will receive

No	Question	Answer		Variable name
1	When you were pregnant with (NAME), did your work involve **<read choice one by one>**?<br><br>Please use the following scale:<br>1 = Never/ Not<br>2 = Rarely<br>3 = Sometimes<br>4 = Often<br>5 = Always<br>98 = Don't know<br>99 No response	1. Manual lifting, carrying, pushing or pulling of loads……………………………….. _____<br>2. Exposure to biological, chemical or physical agents……………………………… _____<br>3. Prolonged periods of sitting or standing _____<br>4. Exposure to extreme temperatures or to vibration…………………………… _____<br>5. Night work (working for at least 2 hours between 22.00 and 05.00)…………… _____<br>6. Extra working time .... _____<br>7. Other hazards/unhealthy conditions (specify) .... _____<br>8. Business trip away from home when pregnant over 7 months……………………… ____		expose
2	Did you request lighter duties or to be transferred to a safer job when you were pregnant with (NAME)?	1. Yes, and my request was accepted<br>2. Yes, but my request was not accepted<br>3. No, I did not request for lighter duties/safer job even I wanted to<br>4. No, I did not request because my current job is safe and suitable.		request
3	Do you think during your pregnancy with (NAME) you were treated unfairly at work in any of the following ways?<br><br>*I will read each option and you choose what situation you fell into?*	1. I was given unsuitable work or workloads	1 Yes<br>0 No	fair
		2. I was moved to a less favorable position in terms of tasks and responsibilities (less senior position)	1 Yes<br>0 No	
		3. I had a reduction in my salary or bonus	1 Yes<br>0 No	
		4. I received a pay rise or bonus that was less than my peers at work	1 Yes<br>0 No	
		5. I received unpleasant comments from my employer and/or colleagues	1 Yes<br>0 No	

		6. I was unfairly criticized or disciplined about my performance at work	1 Yes<br>0 No	
		7. I failed to gain a promotion I felt I deserved or was otherwise sidelined	1 Yes<br>0 No	
		8. I was denied access to training that I would otherwise have received	1 Yes<br>0 No	
		9. I was treated so poorly that I felt I had to leave	1 Yes<br>0 No	
		10. I was dismissed	1 Yes<br>0 No	
		77. Other (specify)__ ___________________	1 Yes<br>0 No	
4	Did you get either paid or unpaid maternity leave when you gave birth to (NAME)?	1. Yes<br>0. No<br>98. Don't know		momoff
5	How many days of leave did you take before giving birth to (NAME)?	_ _ days		before
6	How many days of leave did you take after giving birth to (NAME)?<br><br>*(convert answer to days if given in weeks or months)*	_ _ days		after
7	If you are still on maternity leave, how many **additional days** do you plan to take?	_ _ days		planoff
8	Did anyone else do or is anyone else doing your work while you stopped working for (NAME)'s birth?<br><br>***(Multiple responses possible)***	1. No, nobody<br>2. Yes, colleagues<br>3. Yes, temporary staff<br>4. Yes, boss / leader / owner<br>77. Yes, another person (specify)<br>________________________________<br>98.Do not know<br>99. No response		substitute
9	Did or will you receive any cash benefits because of (NAME)'s birth?	1. Yes, has received<br>2. Yes, not yet received<br>3. Not received → **Move on Q13**		benyes

10	For how many months did or will you receive maternity cash benefits?	_ _ months	benmonth
11	What percentage of your monthly salary/income before (NAME)'s birth did/does the benefit represent?	_ _ %	benper
12	Who provided or is providing the cash benefits?	1. Employer<br>2. Social security<br>3. Local or central government<br>4. Micro-health insurance, community self-help scheme<br>77. Other (specify) ___________	benpay
13	Did your husband/partner get paternity leave when you gave birth to (NAME)? (Ask this question if code of the husband's occupation are 2,3)	1. Yes<br>0. No	dadoff
13b	Is it a paid leave?	1. Yes<br>0. No<br>98. Don't know	
14	How much did the birth of (NAME) affect your household's ability to pay for the most necessary expenses?	1. A great deal<br>2. Much<br>3. Somewhat<br>4. Little<br>5. Not at all	effect
	**Women who have not returned to work: -> move to Question 20**		
15	Did you return to the work before you took maternity leave?	1. Yes<br>0. No, I move on a new job	samework
16	What were the main changes in your <Read each line> that you experienced??	1. Responsibilities<br>a. More responsibilities<br>b. Fewer responsibilities<br>c. Same responsibilities<br>2. Tasks<br>a. More difficult tasks<br>b. Less difficult tasks<br>c. Same tasks	change

		3. Pay<br>a. Higher pay<br>b. Lower pay<br>c. Same pay<br>4. Working time<br>a. More working hours<br>b. Less working hours<br>c. Same working hours	
17	How would you describe your health conditions in the first month when you resumed work after (NAME)'s birth?	1. I felt well healed and recovered from childbirth<br>2. I felt sufficiently healed and recovered from childbirth<br>3. I did not feel sufficiently healed and recovered from childbirth<br>4. I did not at all feel healed and recovered from childbirth<br>98.Do not know<br>99. No response	health
18	Do you get an extra 1 hour off every day to express milk, relax, or breastfeed?	1. Yes<br>0. No	1hour
19	Which factors relating to the workplace have enable a woman in your company to continue breastfeeding her child?<br><br>(***multiple responses possible,*** tick according to mother's words)	1. Mothers can bring the child to work<br>2. Mothers live/lived close to workplace<br>3. Mothers can work/ worked from home<br>4. Mothers can take one or more daily breaks for breastfeeding/milk expression at the workplace<br>5. Mothers can enjoy a reduction in daily working hours to breastfeed at home<br>6. The employer provides / provided a lactation room<br>7. The employer provides / provided daycare or subsidizes the cost for daycare for employees<br>8. There is a childcare facility close to the workplace<br>9. None of the above support factors. Mothers have to try themselves.<br>77. Other (specify)	support

		________________________	
20	According to the Vietnam Labor Code, what are the rights of a working woman who is pregnant or nursing a child under 12 months?<br><br>(Multiple choice, don't read the option, can tick when mentioned a part of the answer)	1. Not to work at night, work overtime, or go on a long-distance travel if 7 months pregnant or more or nursing a child under 12 months.<br>2. No disciplining or terminating the job of a woman during her pregnancy or while nursing a child < 12 months.<br>3. Pregnant women can unilaterally terminate the employment contract, or to temporarily suspend the employment contract during pregnancy with certificate from a competent health care institution that continue working may adversely affect the pregnancy.<br>4. Paid leave during working hour for antenatal care.<br>5. Antenatal care fee covered by health insurance.<br>6. Paid leave for abortion, still birth, family planning, nursing a child < 6 months, caring for sick children < 7 years.<br>7. Paid maternity leave of 6 months (before plus after birth); and extended by one month for each additional child in multiple birth.<br>8. Negotiation about additional unpaid leave with the employer after completion of 6 months paid maternity leave.<br>9. One paid hour for nursing break (rest, express milk, going home to feed the child…)<br>10. Return to the same or at least similar position after maternity leave.<br>11. Received both salary from employer and social insurance if return at 5-6 months (with approval from doctor and mutual agreement with the employer).<br>98. Don't know / did not mention any<br>99. No response	aware

21. Maternity protection policy in Vietnam allows employed women 6 months paid maternity leave and 60-minute breastfeeding breaks a day in the first year after birth.
According to your opinion, what is the effect of of the policy on the following subjects (Variable name: begood**)**

1	2	3	4	5	6
**Very unprofitable**	**Unprofitable**	**Partly unprofitable**	**Partly profitable**	**Profitable**	**Very profitable**

	**Code**
1. Overall benefit for the society	

2. Employers	
3. Mother income	
4. Child health	
5. Mother health	
6. Mother career	
7. My preference on connecting with friends and coworkers	
8. The whole family	

7. Follow up questions

Occationnary, we need to contact respondent to clarify certain information provided or to ask about the interview experience and would need the contact information. The contact information will be used to reach the participant for further information only.

7.1 It is optional, but are you willing to share the best way to follow up with you?<br>0. No-> Thanks the women and stop<br>1. Yes-> fill in the mean of contact	OKcont
7.2 Reviewer self-evaluate if the woman would be a good candidate for IDI<br>0. No-> Thanks the women and stop<br>1. Yes	IDIellig
7.3 In the coming one or two days, we would like to meet you again to learn more about your experiences. It will last for about 45 minutes. Are you willing to participate in the meeting?<br>0. No-> Stop<br>1. Yes-> Read: a colleague of mine will contact you to set up the appropriate date, time, and location for the interview.	IDIagree

THANK YOU VERY MUCH FOR PARTICIPATING IN THIS SURVEY

Notes of the interviewer about the interview session (note_final)
1. Interview venue was quiet
2. No other adults were in the meeting
3. Minimal distraction of children
4. The woman was focus
5. The woman answered correctly
6. No issues relating to the questionnaire
7. No issues relating to the tablet
8. No other issues

Specify (when one of the 8 notes unchecked)

Getting the GEO code outdoor.
